# Causal associations between hypertension and abnormal brain cortical structures: Insights from a bidirectional Mendelian randomization study

**DOI:** 10.1016/j.ijcrp.2024.200354

**Published:** 2024-12-07

**Authors:** Tianxiang Fang, Xizhi Wang, Yingsong Wang, Xiaoya Zheng, Ning Huangfu

**Affiliations:** a Department of Cardiology, The First Affiliated Hospital of Ningbo University, Ningbo, China; b Health Science Center, Ningbo University, Ningbo, China; c Department of Cardiology, Key Laboratory of Precision Medicine for Atherosclerotic Diseases of Zhejiang Province, Ningbo, China; d Clinical Medicine Research Centre for Cardiovascular Disease of Ningbo, Ningbo, China; e Department of Cardiology, Lihuili Hospital Affiliated to Ningbo University, Ningbo, China

**Keywords:** Brain cortical, Caudal anterior cingulate cortex, Heart-brain axis, Hypertension, Insula, Mendelian randomization

## Abstract

**Background:**

Observational studies suggest that hypertension affects brain cortical structure. However, the potential causal association has yet to be entirely determined. Thus, we aim to assess the causality between hypertension and abnormal cortical structure.

**Methods:**

We conducted a bidirectional Mendelian randomization (MR) study to estimate their relationship. Genome-wide association study summary statistics of hypertension (n = 484,598) and brain cortical (surface area and thickness) (n = 51,665) were derived from publicly available databases. Sensitivity analyses were applied to ensure the robustness of the results.

**Results:**

The study showed that hypertension was associated with a decline in total brain cortical thickness [β, -0.0308 mm; 95 % confidence interval (CI), -0.0610 to -0.0007; *p* = 0.045] and the insula thickness [β, -0.0415 mm; 95 % CI, -0.0772 to -0.0057; *p* = 0.023]. A null association was observed between hypertension and other brain regions. In the reverse MR analysis, the total cortical surface area (per 1 SD increase) significantly decreased the incidence of hypertension [odds ratio (OR), 0.976; 95 % CI, 0.963 to 0.990; *p* = 5.15E-04]. The caudal anterior cingulate cortex thickness (per 1 SD increase) was significantly associated with an increased risk of hypertension [OR, 1.057; 95 % CI, 1.034 to 1.082; *p* = 1.08E-06]. Moreover, we found several nominally associated gyri, including cuneus, isthmus cingulate, middle temporal, para hippocampal, posterior cingulate, superior temporal, and medial orbitofrontal, influence the incidence of hypertension.

**Conclusion:**

Our study showed causal relationships between hypertension and changes in specific brain cortical, providing new evidence for the heart-brain axis theory.

## Highlights

• The potential causal association between hypertension and specific brain structure change remains unclear.
• Hypertension may lead to a decline in total brain cortical thickness and insula thickness.
• Similarly, alterations in certain cortical regions also influence the incidence of hypertension.
• Our study provides comprehensive and strong evidence for the theory of the heart-brain axis.

## Graphical abstract

**Figure:**
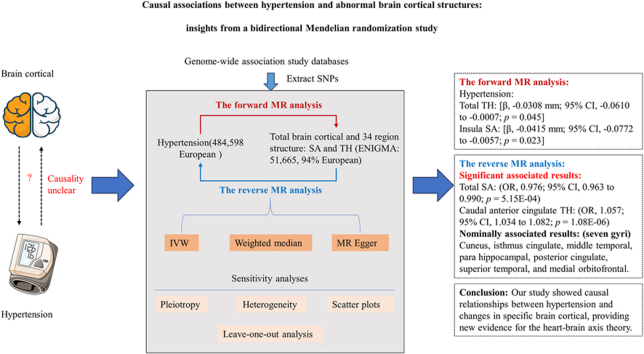

## 1 Introduction

Neurocardiology is an emerging specialty focusing on the intricate interaction between the brain and heart [1]. Byer et al. first reported in 1947 that cerebral vascular disease can induce myocardial damage and arrhythmia [2]. Since then, a growing body of evidence has shown that multitudinous brain injuries can cause cardiac dysfunction, arrhythmias and heart failure, such as stroke and traumatic brain injury [1,3]. Furthermore, cardiovascular diseases such as coronary heart disease, hypertension and atrial fibrillation also increase the incidence of stroke [1,4].

Hypertension is one of the most common cardiovascular diseases, and normal blood pressure is pivotal in maintaining cerebral perfusion [5,6]. The brain is considered to be an end-organ of hypertension and is damaged late in life by stroke or dementia [6]. Emerging evidence, however, shows that brain structure and function deficits relate to increased but prehypertensive blood pressure and may be altered before diagnosis of hypertension [7]. Notably, the incidence of hypertension was higher in the older population, who often experience brain atrophy and cognitive decline accompanying age-related change [8]. Traditional observational studies were unpersuasive due to potential confounding factors and reverse causality bias. Therefore, it is unclear whether a genetic relationship exists between hypertension and abnormalities in cortical structures.

Randomized controlled trials (RCTs) face challenges in clinical implementation due to multiple limiting factors. At the same time, observational, experimental approaches may yield biased study outcomes due to confounding variables and reverse causation, ultimately diminishing their credibility. Mendelian randomization (MR) employs genetic variability as a randomized tool for studying the causal relationship between risk factors associated with outcomes [9]. Specifically, the natural random classification of genetic variation during meiosis, typically single-nucleotide polymorphisms (SNPs), produces a random distribution of genetic variation in a population [10,11]. Because these genetic variants are commonly independent of confounders, differences in the outcomes between variant carriers and non-carriers can be attributed to differences in risk factors. Thus, we conducted a bidirectional MR study to estimate the causality between hypertension and alterations in brain cortical structure.

## 2 Materials and methods

### 2.1 Study design

**Fig. 1:**
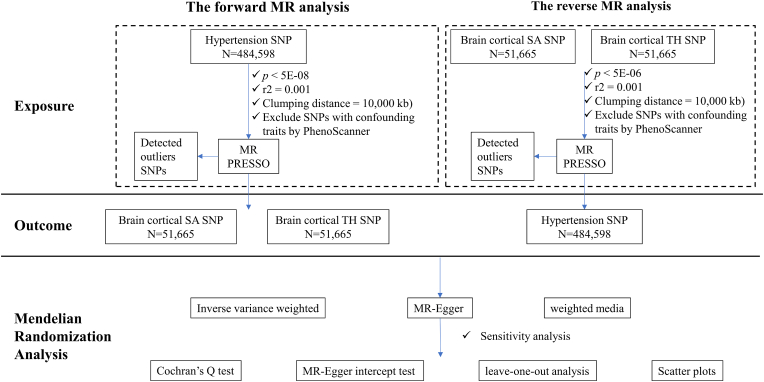
**Diagram of the bidirectional Mendelian randomization study for the causal relationship between hypertension and alteration in brain cortical structure.** MR, mendelian randomization; SNP, single nucleotide polymorphism; SA, surface area; TH, thickness; MR-PRESSO, MR pleiotropy residual sum and outlier.

Bidirectional MR analysis was utilized to explore causal relationships between hypertension and abnormal changes in brain cortical structure. SNPs from genome-wide association study (GWAS) were applied as instrumental variables (IVs) to estimate the causal relationship in MR analysis. Criteria for screening IVs included: (1) SNPs were strongly linked to exposure factors; (2) SNPs were not associated with potential confounders; (3) SNPs influenced the outcome only through exposure, not directly related to outcomes. The bidirectional MR design flow is presented in Fig. 1.

### 2.2 The data source for hypertension and brain cortical structure

The GWAS dataset for hypertension was derived from IEU Open GWAS, including 129,909 cases and 354,689 controls of European ancestry [12]. GWAS summary statistics of brain cortical structure were provided by the Enhancing Neuroimaging Genetics through Meta-Analysis (ENIGMA) consortium [13]. The ENIGMA consortium encompassed 51,665 individuals from 60 cohorts worldwide, approximately 94 % of which were European origination. This study analyzed the surface area (SA) and average thickness (TH) of the total brain cortical and the SA and TH of 34 regions defined by the Desikan–Killiany atlas [14]. These data are available at the website https://enigma.ini.usc.edu (Table 1).

**Table 1:** Details of the GWAS data used for Mendelian randomization analysis.

Phenotype	Ethnicity	Sample size	nSNPs	Year	GWAS ID
Hypertension	European	484,598	9,587,836	2021	ebi-a-GCST90038604
Brain cortical surface area	European	51,665	8,567,615	2020	NA
Brain cortical thickness	∼94 % European	51,665	8,619,054	2020	NA

SNP, single nucleotide polymorphism; GWAS, genome-wide association study.

### 2.3 Selection of genetic IVs

We used SNPs with genome-wide significance (*p* < 5E-08) as IVs to estimate the causal effects of hypertension on brain cortical structure. In the reverse MR analysis, SNPs with *p* < 5E-06 were selected to estimate the causal estimation of brain cortical structure on hypertension [9]. Additionally, linkage disequilibrium (LD) clumping (r2>0.001 and < 10,000 kb) was used to ensure the independence of each IV. To minimize correlated pleiotropy, we detected the SNPs that were associated with confounder traits through PhenoScanner, including basic information (smoke, alcohol consumption, medication use, educational years, and age); body-related indicators (body mass index, waist-hip ratio, weight, height, and obesity); metabolism-related indexes (diabetes, HbA1c levels, low-density lipoprotein, high-density lipoprotein, and triglyceride); mental illness and cerebrovascular diseases (schizophrenia, depression, anxiety, worrier tendencies, neuroticism, autism, insomnia, and stroke); autoimmune diseases (multiple sclerosis, rheumatoid arthritis, Crohn disease, and ulcerative colitis); other confounders factors (thyroid disease and chronic kidney disease) and their respective related-confounder factors [[15], [16], [17], [18], [19]]. Finally, the F-statistic for each SNP was calculated to assess the strength of IVs. The F-statistic less than 10 indicated that the SNP was weak; therefore, IVs would be removed.

### 2.4 MR analysis and sensitivity analysis

In this study, the inverse-variance weighted (IVW) (fixed effects and random effects) method was considered the primary statistical method due to its highest precision [20]. Moreover, two other different MR methods (MR-Egger and weighted media) were performed to complement and improve the robustness of the results. MR-Egger regression provided a consistent estimate of the causal effect in the presence of horizontal pleiotropy. The weighted median method offered reliable causal estimates when less than half of the SNPs were effective IVs [20].

To improve the reliability of the genetic instruments, we conducted Cochran's Q test to identify heterogeneity. MR-Egger intercept test was used to assess horizontal pleiotropy. Moreover, we attempted to mitigate horizontal pleiotropy by utilizing the MR pleiotropy residual sum and outlier (MR-PRESSO) analysis to detect outlier SNPs before conducting the final MR analysis. Lastly, the leave-one-out analysis was performed to check whether a single SNP influenced the primary causal relationship.

### 2.5 Statistics

To calculate the effect size per 1 SD change in the causal estimation of brain cortical structure on hypertension, we converted each β-coefficient and corresponding SE, shown in the original GWAS, to SD units [21]. Additionally, we used a Bonferroni method to correct the *p*-value threshold to address the issue of multiple testing, defined as *p* < 0.05/2 × n (n represented the number of outcomes) [20]. Thus, a *p* < 0.0125 (0.05/2 × 2) for significant results in the estimation of SA and TH of total cortical, while a *p* < 3.68E-04 (0.05/2 × 68) for significant results in the estimation of SA and TH of the specific brain region. The *p* < 0.05 was considered nominally significant. All analyses in this study were completed in R software (version 4.3.2).

## 3 Results

The detailed characteristics of the SNPs associated with hypertension and brain cortical structure are displayed in Table S1. The F-statistics of all SNPs were higher than the threshold of 10, indicating no evidence of weak instruments.

### 3.1 The causal effect of hypertension on brain cortical structure

**Fig. 2:**
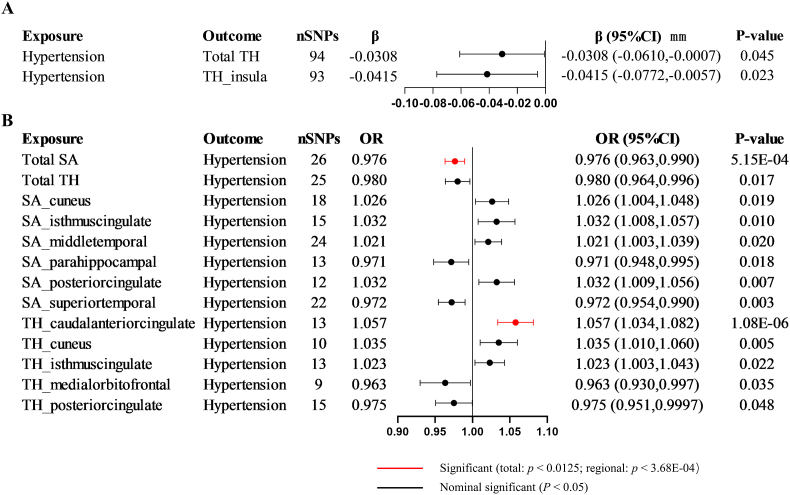
**The significant and nominally significant causalities in the bidirectional Mendelian randomization analysis.** OR, odds ratio; CI, confidence interval; SNP, single nucleotide polymorphism; SA, surface area; TH, thickness.

**Fig. 3:**
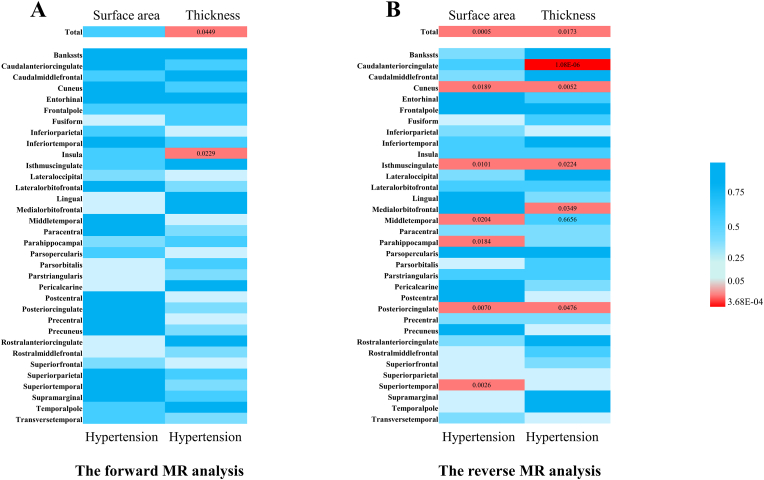
**IVW estimates the causal relationship between hypertension and brain cortical structure.** (A) represent the causal effect of hypertension on cortical structure. (B) represent the causal effect of cortical structure on hypertension. The colour of each block represents the *p*-value of every MR analysis. The red blocks indicate a *p*-value <0.05, and the blue blocks indicate a *p*-value ≥0.05. At the global level, a *p*-value <0.0125 is considered significant, whereas a *p*-value <3.68E-04 is considered significant at the regional level. The *p*-value lesser than 0.05 is considered nominally significant.

At the global level, genetically predicted hypertension was associated with a decline in total brain cortical TH [β, -0.0308 mm; (95 % confident interval [CI]), -0.0610 to -0.0007; *p* = 0.045] but no causal relationship with SA (*p* = 0.575). At the regional level, hypertension decreased TH of the insula [β, -0.0415 mm; 95 % CI, -0.0772 to -0.0057, *p* = 0.023] (Fig. 2 and Table S2). The heatmaps illustrating the IVW-derived *p*-values for hypertension on brain cortical structure are shown in Fig. 3A.

### 3.2 The causal effect of brain cortical structure on hypertension

In the reverse MR, we identified several significant or nominally significant gyrus influences on the incidence of hypertension (Fig. 2 and Table S3). At the global level, the total cortical SA (per 1 SD increase) significantly decreased the risk of hypertension [OR, 0.976; 95 % CI, 0.963 to 0.990; *p* = 5.15E-04]. Additionally, the cortical TH (per 1 SD increase) was nominally associated with the risk of hypertension decline [OR, 0.980; 95 % CI, 0.964 to 0.996; *p* = 0.017]. On the contrary, the caudal anterior cingulate cortex (ACC) TH (per 1 SD increase) was significantly associated with an increased risk of hypertension [OR, 1.057; 95 % CI, 1.034 to 1.082; *p* = 1.08E-06]. Furthermore, several nominally associated gyri, including cuneus SA, isthmus cingulate SA, middle temporal SA, para hippocampal SA, posterior cingulate SA, superior temporal SA, cuneus TH, isthmus cingulate TH, medial orbitofrontal TH, and posterior cingulate TH, showed potentially influences on hypertension. The heatmaps illustrating the IVW-derived *p*-values for brain cortical structure on hypertension were shown in Fig. 3B.

### 3.3 Sensitivity analysis

All p-values of MR Egger intercept tests of the above positive results were >0.05 except for middle temporal SA on hypertension, indicating no horizontal pleiotropy existed (Table 2). However, Cochrane's Q test suggested that some results suffered from different degrees of heterogeneity (Table 2). We used the random-effects IVW as the final results for these results [19]. The scatter plots were displayed in (Figs. S1–S4). Furthermore, the leave-one-out analysis indicated that all estimates were robust, as removing any SNPs did not result in a significant alteration (Figs. S5–S8).

**Table 2:** Heterogeneity and pleiotropy tests of the significant and nominally significant Mendelian randomization estimates.

Exposure	Outcome	nSNPs	IVW P-value	Cochrane's Q test		MR−Egger intercept test
				MR-egger	IVW test	
Hypertension	Total TH	94	0.045	0.199	0.218	0.736
Hypertension	TH_insula	93	0.023	0.355	0.147	0.668
Total SA	Hypertension	26	5.15E-04	0.385	0.419	0.539
Total TH	Hypertension	25	0.017	0.126	0.089	0.154
SA_cuneus	Hypertension	18	0.019	0.056	0.024	0.116
SA_isthmuscingulate	Hypertension	15	0.010	0.072	0.096	0.745
SA_middletemporal	Hypertension	24	0.020	0.301	0.093	0.018
SA_parahippocampal	Hypertension	13	0.018	0.091	0.127	0.908
SA_posteriorcingulate	Hypertension	12	0.007	0.146	0.191	0.73
SA_superiortemporal	Hypertension	22	0.003	0.071	0.093	0.959
TH_caudalanteriorcingulate	Hypertension	13	1.08E-06	0.655	0.671	0.411
TH_cuneus	Hypertension	10	0.005	0.499	0.533	0.442
TH_isthmuscingulate	Hypertension	13	0.022	0.89	0.898	0.455
TH_medialorbitofrontal	Hypertension	9	0.035	0.031	0.046	0.697
TH_posteriorcingulate	Hypertension	15	0.048	0.016	0.009	0.222

SNP, single nucleotide polymorphism; SA, surface area; TH, thickness.

## 4 Discussion

Using a bidirectional MR study, our study investigated the causal relationships between hypertension and brain cortical structure. The results showed that hypertension could decrease global cortical TH and the insula TH. Reverse MR results showed that genetically predicted global cortical SA and the caudal ACC TH were significantly associated with the risk of hypertension. Meanwhile, we also found that several cortical regions would influence the development of hypertension. Our findings provided a more comprehensive understanding of the interactions between hypertension and brain structure.

Evidence from cross-sectional and longitudinal studies showed that individuals with hypertension exhibited total and regional cortical atrophy compared to those without hypertension [5,22]. Besides, a meta-analysis involving 26 observational studies of 1710 individuals indicated that hypertension and higher blood pressure levels were closely associated with total or regional brain volume reduction [23]. Our findings are consistent with these observational studies, which show that hypertension leads to cortical thinning. The cortical TH reduction was a sensitive risk factor for cognitive impairment and Alzheimer's disease conversion [5]. Mechanically, hypertension contributed to micro- and macro-vascular injury, including endothelial dysfunction, atherosclerosis, and vascular narrowing. This impaired cerebral blood flow autoregulation in the brain [5]. Reduced brain perfusion correlated with decreased brain structure integrity [24]. Additionally, hypertension-induced oxidative stress, inflammation and intervention of the innate immune system further impaired the structure and function of cerebral blood vessels. It ultimately mediated hypertensive brain damage progression, containing cerebrovascular diseases and cognitive impairment [25]. Our study indicates that patients with hypertension should pay attention to alterations in cortical structure, especially the insula.

In addition, we also found that brain structural abnormalities were associated with hypertension. Reverse MR analysis showed a negative correlation between the SA and TH of global cortical with hypertension, which might partially account for the higher risk of hypertension in older individuals since global brain atrophy was commonly seen with advancing age [26]. Over-driven sympathetic nerve activity (SNA) is known to be one of the critical mechanisms of hypertension [27,28]. It has been demonstrated that altered autonomic nervous system (ANS) for blood pressure regulation with ageing, including SNA elevation and cardiac vagal inhibition reduction [29]. In addition to changes in ANS with ageing, there is evidence of alteration of the brain's non-neural cells. Astrocytes play an essential role in the cellular homeostasis of the brain, which will be dysfunctional along with ageing [26]. A study found that the phenotypes of astrocytes shifted to inflammatory states in advancing ageing [30]. These ageing astrocytes might impact the activity of the SNA by secreting pro-inflammatory cytokines such as C-X-C motif chemokine ligand 10 [26,30].

Interestingly, unlike the global cortical structure, the increased TH in caudal ACC resulted in a higher risk of hypertension. ACC is a critical region for the interactions of cognitive control, emotional processing, and vagal modulation [31]. Iceta et al. pointed out that lower grey matter density in the caudal ACC was linked with higher visceral adiposity index and metabolic alterations (such as insulin resistance and type 2 diabetes) [32]. Recently, an MR study showed that TH of the caudal ACC positively correlated to higher carbohydrate intake [9]. A chronic diet high in sugar and fat accelerated the development of metabolic syndrome and hypertension [33]. Moreover, the caudal of ACC was involved in the appraisal and expression of negative emotion [34]. A prospective cohort (median = 12 years) showed that parent-rated negative emotionality was associated with more significant TH of the caudal ACC bilaterally [35]. These negative emotions, including depression, anger, hostility, and anxiety, have been identified as significant causes of hypertension [36]. Moreover, the study showed thicker left caudal ACC in major depressive disorder subjects as compared to controls [37]. Notably, negative emotions might also be a trigger of uncontrolled eating or binge eating behaviors [32]. Conversely, several previous observational studies reported that the caudal region of ACC TH was positively associated with resting heart rate variability (HRV), an index of vagal modulation of cardiac activity [31]. Low HRV was independently associated with the risk of hypertension [31]. Thus, it is plausible to speculate that the caudal ACC influences elevated blood pressure through cognitive control changes and negative emotions rather than vagal modulation dysfunction. In summary, our study suggested that an increase in the caudal ACC TH resulted in a vulnerability to hypertension.

**Fig. 4:**
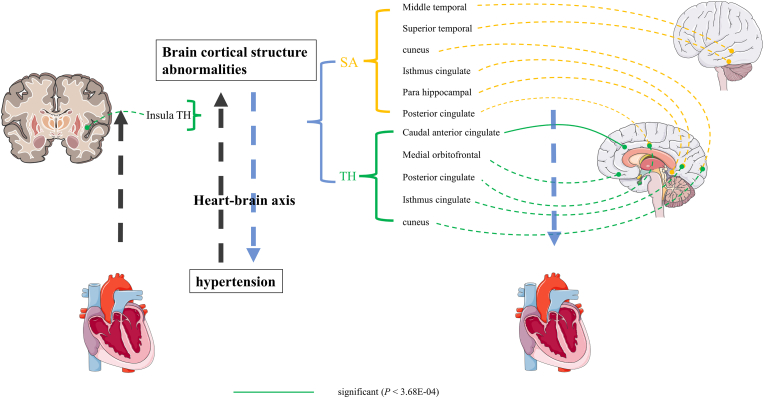
**Using a framework for visualizing the results of bidirectional Mendelian randomization analysis, we reveal the connections of hypertension and brain cortical structure alteration, providing new evidence for the theory of heart-brain axis.** SA, surface area; TH, thickness.

Remarkably, caution should be exercised when interpreting other nominal significance (IVW-derived *p* < 0.05). These regions’ structural alterations might be a precursor to functional alterations, leading to dysregulation of blood pressure regulation. Our results showed a direct causal relationship between hypertension and specific cortical structure. This result supports the interaction between the heart and the brain, providing comprehensive and robust evidence for the theory of neurocardiology (Fig. 4)**.**

Our study had some important strengths. The primary strength of our study was a large-scale MR analysis that overcame the shortcomings of observational studies. The findings provided new evidence on the causal relationship between hypertension and specific cortical changes, giving new inspirations about the heart-brain axis. It was essential to provide more optimized surveillance and treatment for patients with hypertension. However, there were also limitations to consider. First, our study populations were almost derived from European cohorts, which might restrict the generality of our results. Second, we selected the SNP with *p* < 5E-06 rather than the traditional GWAS significance threshold of *p* < 5E-08 to intake more genetic variations as IVs for brain cortex structure. Lastly, the specific mechanisms between hypertension and brain structure remained unclear, warranting further prospective verification and mechanism exploration in future studies.

## 5 Conclusion

In conclusion, this bidirectional MR study yields compelling evidence for the causal relationship between hypertension and brain cortical structure changes. Our results indicated the significance of wide attention and examination of potential changes in the corresponding brain region structure and function for patients with hypertension, i.e. total brain cortical and the insula. Furthermore, we identified several significant or nominally significant gyrus that influenced the incidence of hypertension. To some extent, this study provides new evidence for the theory of neurocardiology and the heart-brain axis. However, the biological mechanisms underlying hypertension and brain cerebral structure interaction require further elucidation.

## CRediT authorship contribution statement

**Tianxiang Fang:** Writing – original draft, Supervision, Software, Investigation, Conceptualization. **Xizhi Wang:** Writing – original draft, Supervision, Software, Investigation, Conceptualization. **Yingsong Wang:** Writing – original draft, Visualization, Validation, Resources. **Xiaoya Zheng:** Writing – review & editing, Validation, Resources, Data curation. **Ning Huangfu:** Writing – review & editing, Supervision, Software, Methodology, Investigation, Conceptualization.

## Availability of data and materials

In this research, we utilize publicly available GWAS summary data. The GWAS dataset for hypertension was derived from IEU Open GWAS (https://gwas.mrcieu.ac.uk/). The brain cortical structure summary data are available on the Enhancing Neuroimaging Genetics through Meta-Analysis (ENIGMA) websites (https://enigma.ini.usc.edu/).

## Ethics declarations

Ethics approval and consent to participate: Not applicable.

Consent for publication: Not applicable.

## Disclosures

The authors declare that the research was conducted in the absence of any commercial or financial relationships that could be construed as a potential conflict of interest.

## Sources of funding

This work was supported by the 10.13039/501100001809National Natural Science Foundation of China (82200489), Ningbo Young Scientific and Technological Innovation Leading Talent Program (2024QL027).
