# Supplementary material for: Causal associations between hypertension and abnormal brain cortical structures: Insights from a bidirectional Mendelian randomization study
Source: Int J Cardiol Cardiovasc Risk Prev. 2024 Dec 7;24:200354. doi: 10.1016/j.ijcrp.2024.200354 (PMC11696852; doi:10.1016/j.ijcrp.2024.200354)
Supplement: Multimedia component 1 [file mmc1.docx]

**Supplementary Figures**

**Figure S1.** Scatter plots of significant results from genetically predicted brain cortical structures on hypertension.

**Figure S2.** Scatter plots of nominal significant results from genetically predicted hypertension on brain cortical structures.

**Figure S3.** Scatter plots of nominal significant results from genetically predicted brain cortical SA on hypertension.

**Figure S4.** Scatter plots of nominal significant results from genetically predicted brain cortical TH on hypertension.

**Figure S5.** Leave-one-out analysis of significant results from genetically predicted brain cortical structures on hypertension.

**Figure S6.** Leave-one-out analysis of nominal significant results from genetically predicted hypertension on brain cortical structures.

**Figure S7.** Leave-one-out analysis of nominal significant results from genetically predicted brain cortical SA on hypertension.

**Figure S8.** Leave-one-out analysis of nominal significant results from genetically predicted brain cortical TH on hypertension.


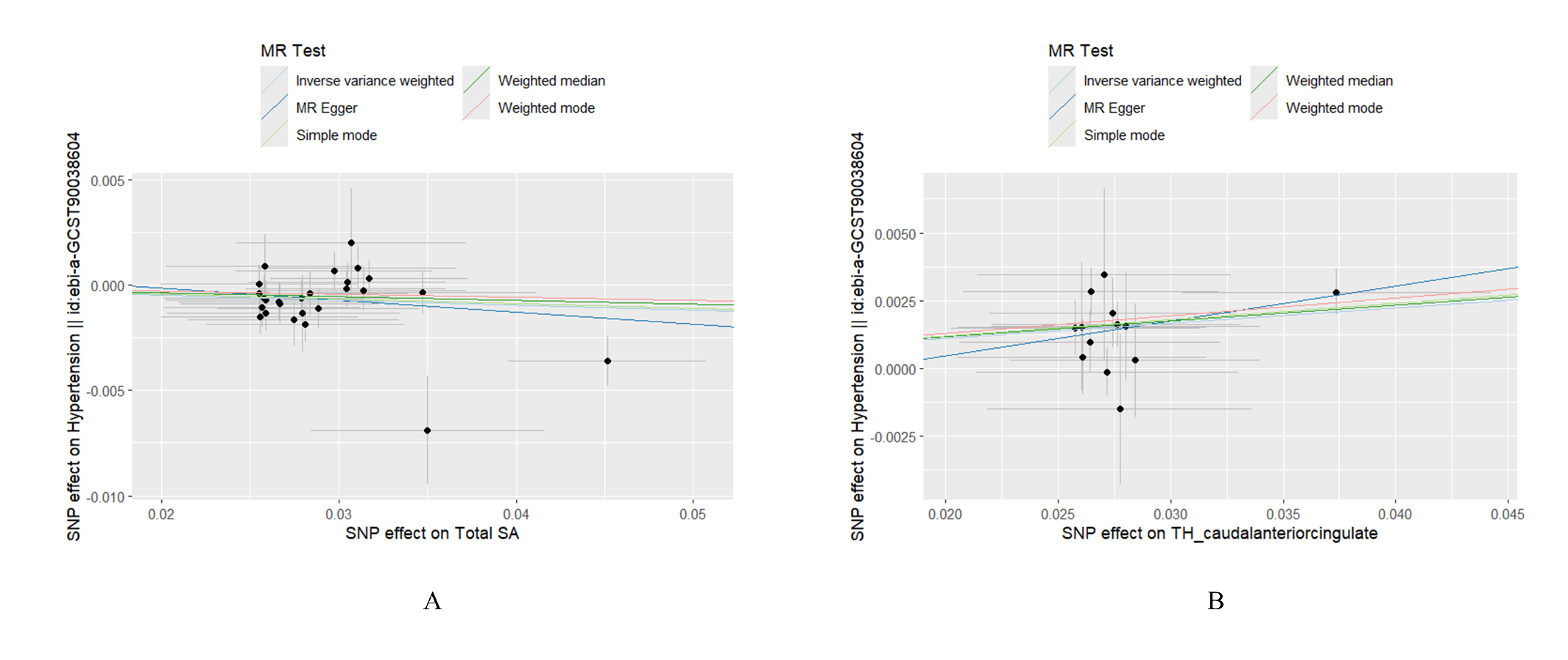
**Figure S1.** Scatter plots of significant results from genetically predicted brain cortical structures on hypertension. (A) Total cortical SA; (B) TH of caudal anterior cingulate. SA, surface area; TH, thickness.


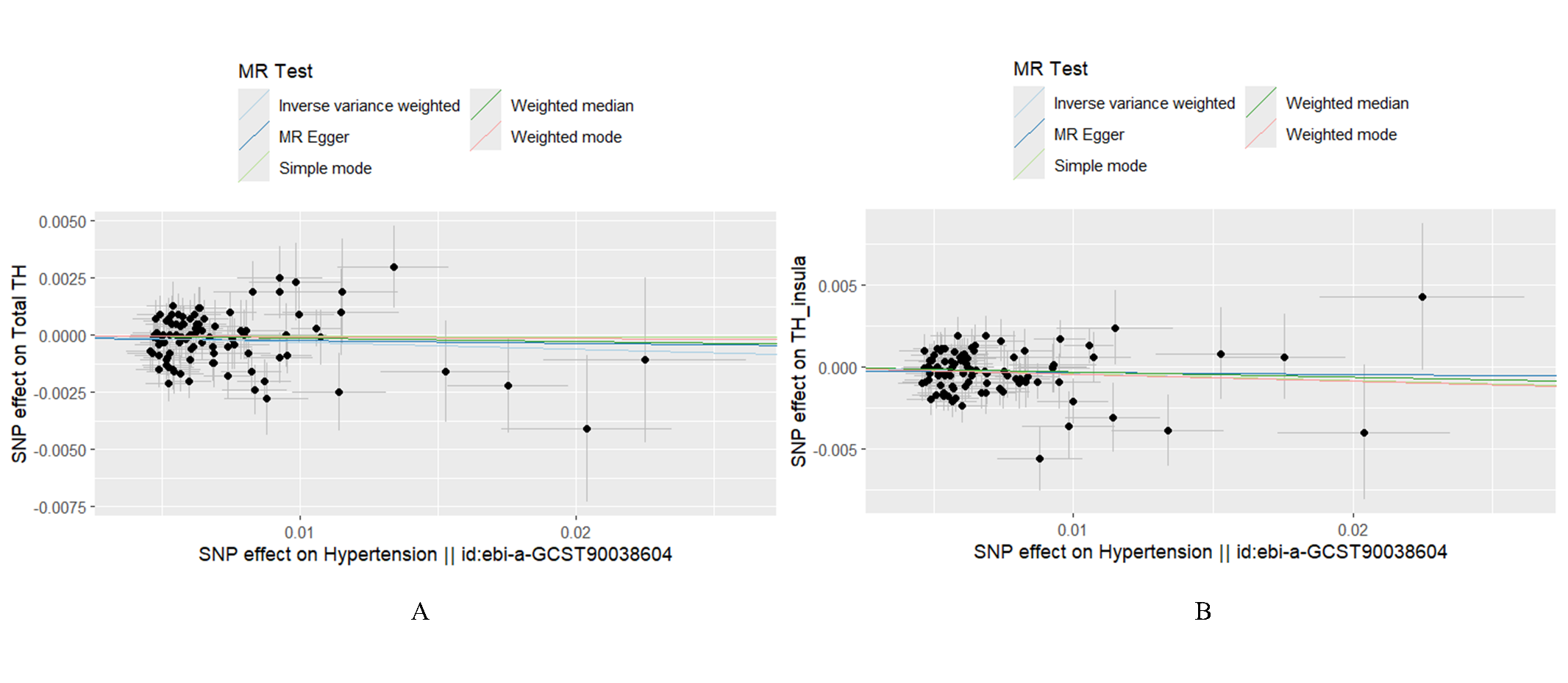


**Figure S2.** Scatter plots of nominal significant results from genetically predicted hypertension on brain cortical structures. (A) Total cortical TH; (B) TH of insula. SA, surface area; TH, thickness.


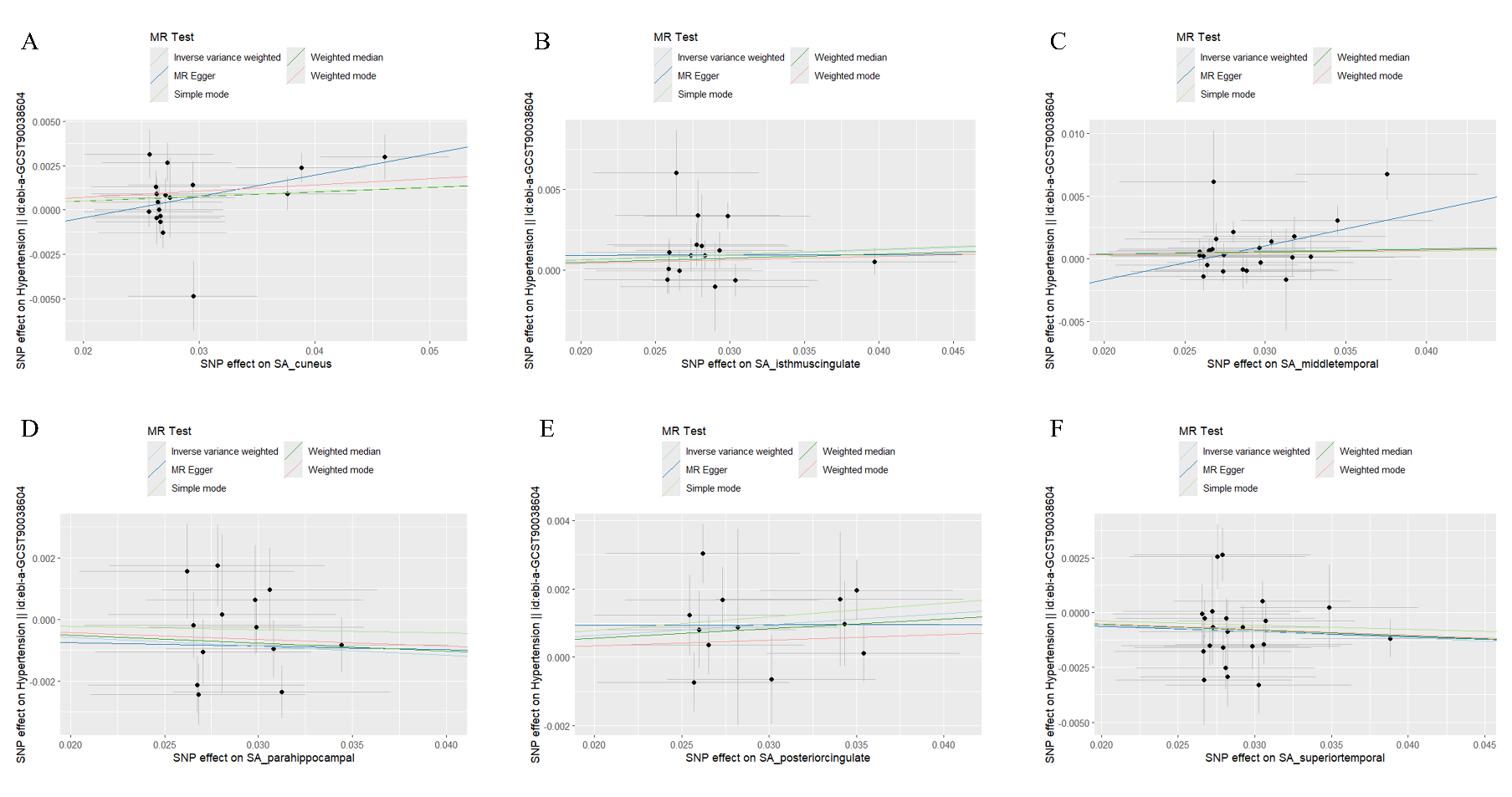


**Figure S3.** Scatter plots of nominal significant results from genetically predicted brain cortical SA on hypertension. (A) SA of cuneus; (B) SA of isthmus cingulate; (C) SA of middle temporal; (D) SA of para hippocampal; (E) SA of posterior cingulate; (F) SA of superior temporal. SA, surface area.


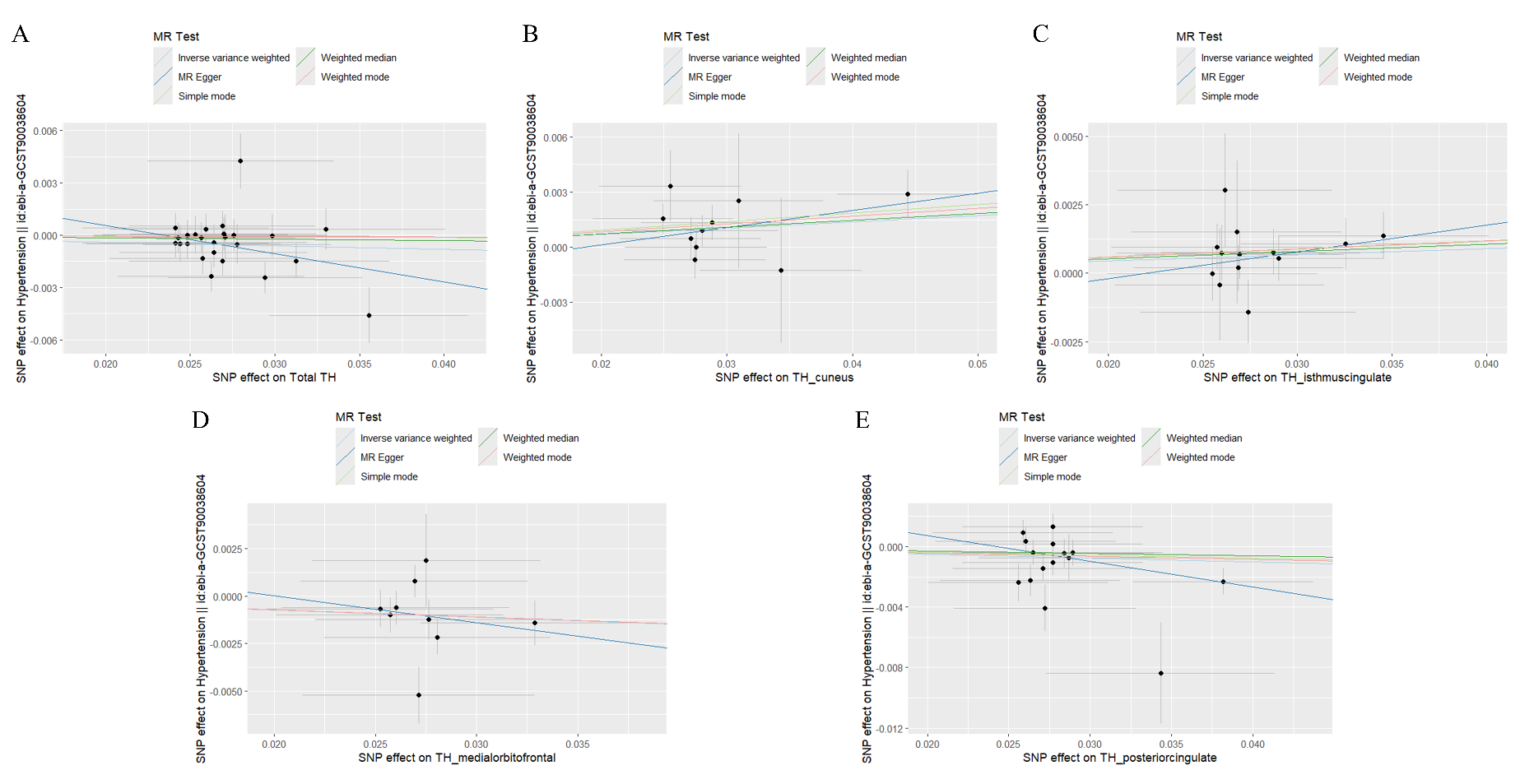


**Figure S4.** Scatter plots of nominal significant results from genetically predicted brain cortical TH on hypertension. (A) Total cortical TH; (B) TH of cuneus; (C) TH of isthmus cingulate; (D) TH of medial orbitofrontal; (E) TH posterior cingulate. TH, thickness.


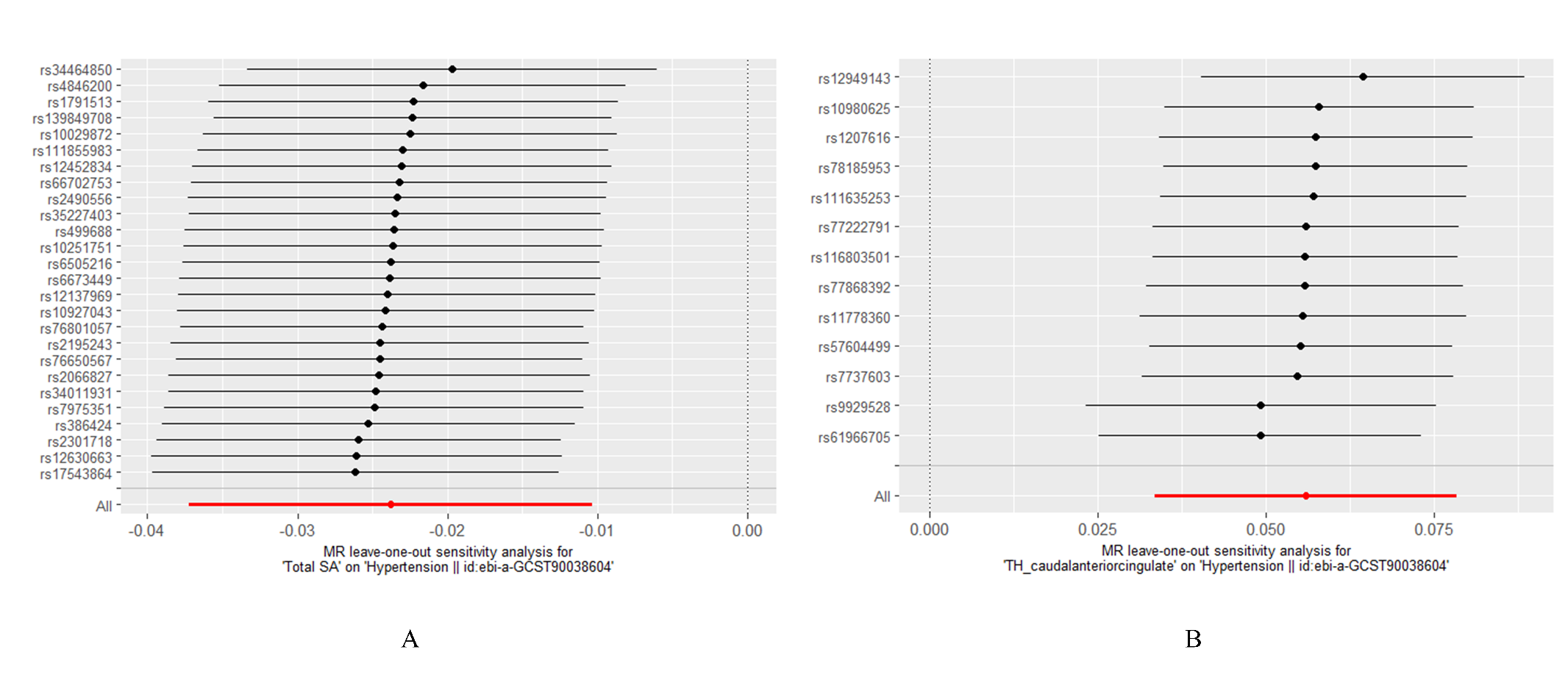
**Figure S5.** Leave-one-out analysis of significant results from genetically predicted brain cortical structures on hypertension. (A) Total cortical SA; (B) TH of caudal anterior cingulate. SA, surface area; TH, thickness.


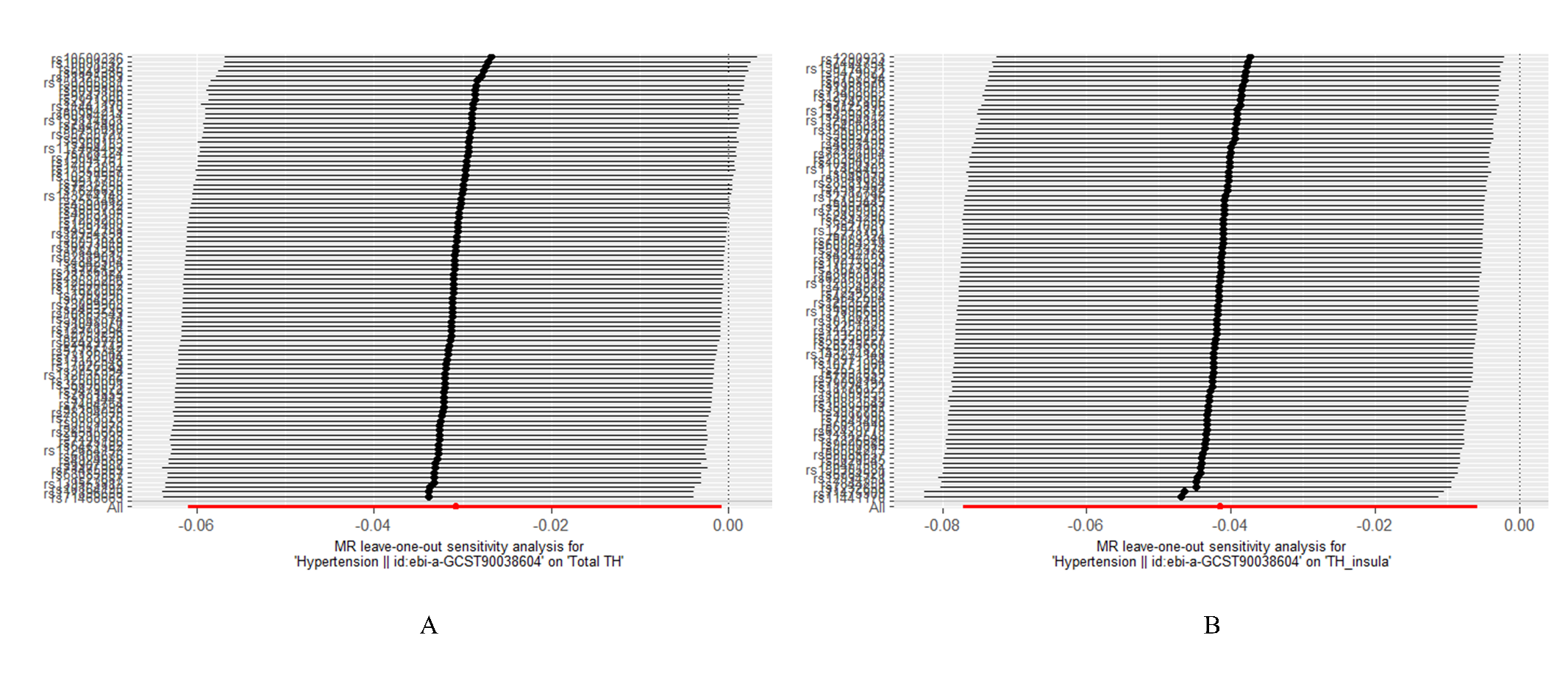


**Figure S6.** Leave-one-out analysis of nominal significant results from genetically predicted hypertension on brain cortical structures. (A) Total cortical TH; (B) TH of insula. SA, surface area; TH, thickness.


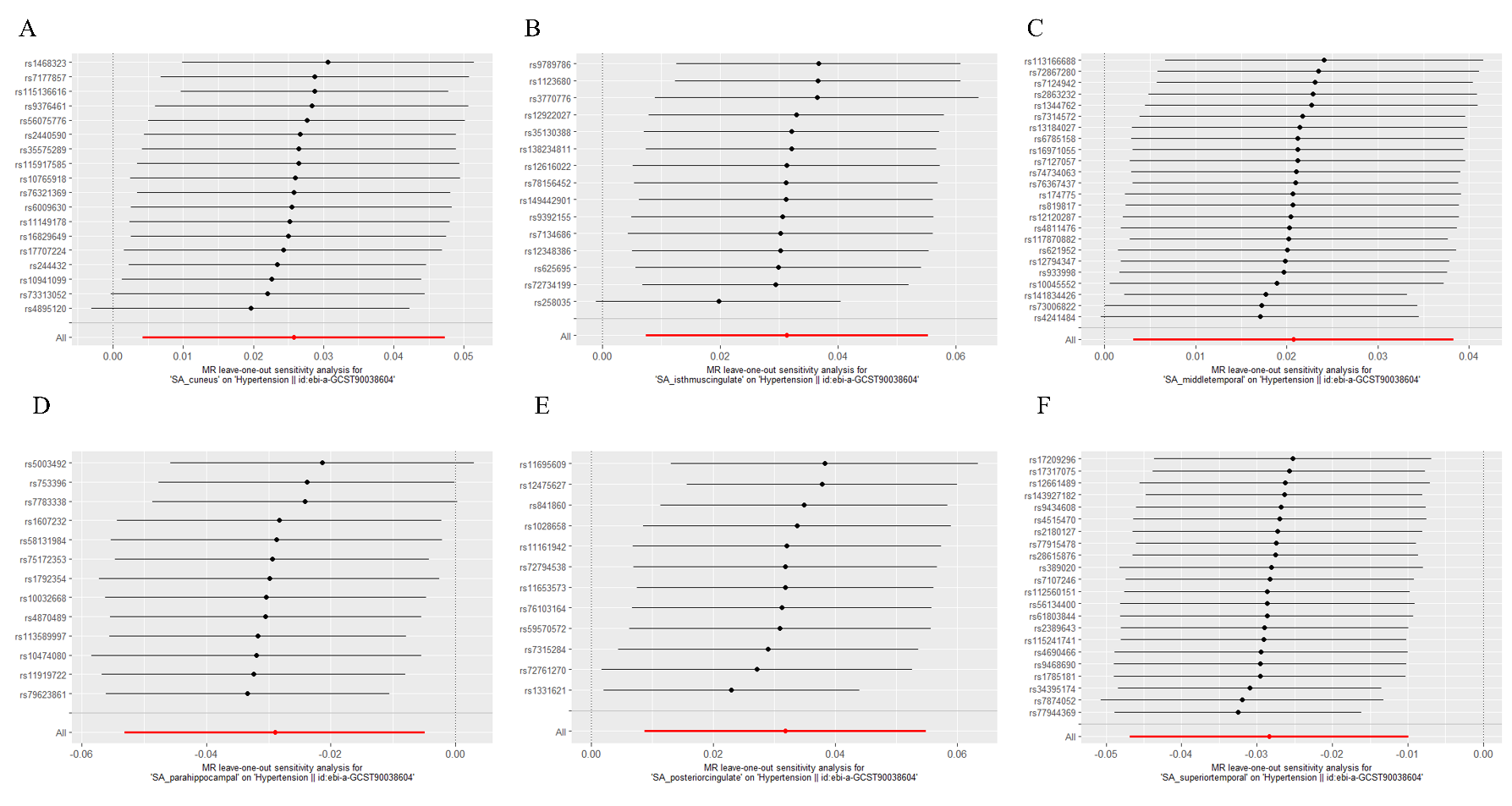


**Figure S7.** Leave-one-out analysis of nominal significant results from genetically predicted brain cortical SA on hypertension. (A) SA of cuneus; (B) SA of isthmus cingulate; (C) SA of middle temporal; (D) SA of para hippocampal; (E) SA of posterior cingulate; (F) SA of superior temporal. SA, surface area.


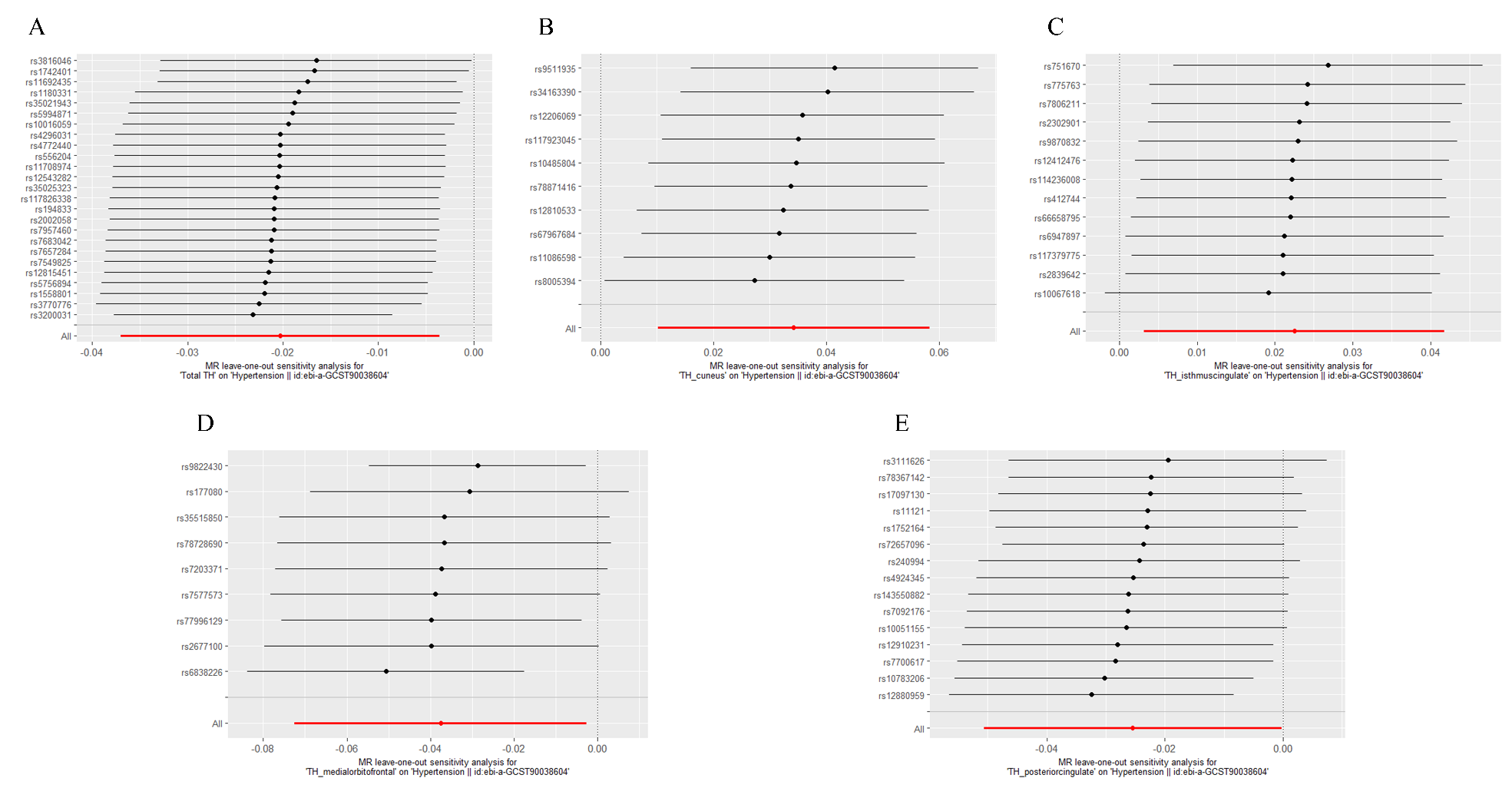


**Figure S8.** Leave-one-out analysis of nominal significant results from genetically predicted brain cortical TH on hypertension. (A) Total cortical TH; (B) TH of cuneus; (C) TH of isthmus cingulate; (D) TH of medial orbitofrontal; (E) TH posterior cingulate. TH, thickness.
